# Supplementary material for: TEP-1, a glial thioester protein is required for cilia organization and intraflagellar transport in ensheathed sensory neurons
Source: bioRxiv. 2025 Oct 24:2025.10.23.684198. Preprint. [Version 1] doi: 10.1101/2025.10.23.684198 (PMC12633222; doi:10.1101/2025.10.23.684198)
Supplement: 1 [file NIHPP2025.10.23.684198V1-supplement-1.pdf]

bioRxiv preprint doi: https://doi.org/10.1101/2025.10.23.684198; this version posted October 24, 2025. The copyright holder for this preprint (which was not certified by peer review) is the author/funder, who has granted bioRxiv a license to display the preprint in perpetuity. It is made available under aCC-BY 4.0 International license.

Figure S1.

A, Comparison of the modular structures of *TEP-1 splice variant secreted form (TEP-1S) and GPI anchored form (TEP-1GPI), CD109, C3, C4, C5 and alpha- 2-macroglobulin (based on 21).* Conserved domain information was compiled from Interpro, SMART domain architecture analysis, and NCBI Web CD-Batch Search Tool. Abbreviations are as follows: MG2:Macroglobulin domain, A2M_BRD: Alpha-2-macroglobulin bait region domain, A2M: Alpha-2-macroglobulin family, ANATO: Anaphylatoxin homologous domain, Ted_complement: complement thioester domain, A2M_rec: Alpha-2-macroglobulin receptor domain, C345C: NTR (netrin) module. Arrow shows location of CRISPR mediated insertion of mNeon Green.

B, *tep-1 expression by CEP sheath and phasmid socket glia.* GFP expressed under the control of *tep-1* 5' untranslated regulatory sequence overlaps with myrRFP under the control of ptr-10 5'

bioRxiv preprint doi: https://doi.org/10.1101/2025.10.23.684198; this version posted October 24, 2025. The copyright holder for this preprint (which was not certified by peer review) is the author/funder, who has granted bioRxiv a license to display the preprint in perpetuity. It is made available under aCC-BY 4.0 International license.

regulatory sequences in CEPsh and Phso glial cells in young adult animal. Anterior is oriented left. Scale bars is 5μm.

C, Confocal image of TEP-1::mNG translational fusion in tandem with VAB-9::wrmScarlet in vulval and seam epithelial cells and TEP-1::mNG in uterine fluid surrounding a fertilized egg (arrows). Scale bar is 5μm. Dorsal is oriented up.

Figure S2.

A, Comparison of CFH-1::GFP and inversin/NPHP-2::mCherry localization in CEP neurons of WT and *tep-1* mutant day 1 adults. Right, scatter plots of inversin/NPHP-2 length in CEP neurons of day 1 adult animals. Error bars indicate 95% confidence intervals. Significance is indicated by brackets with asterisks as follows: ** P<.01. Scale bar is 2.5μm.

Figure S3. *Comparison of anterograde movement of IFT88/OSM-5,* dynein heavy chain CHE-3, and CHE-11/IFT140 in *WT and tep-1 mutant C. elegans day 4 adults.*

A, Plots of anterograde IFT velocity vs. distance from cilia base for IFT88/OSM-5::GFP (top), dynein heavy chain CHE-3, and CHE-11/IFT140 (bottom) in phasmid sensory neurons of WT(black) and *tep-1* mutant (yellow) animals. Note that in *tep-1* mutant animals IFT velocities are slightly faster than WT in anterograde IFT. B, Representative anterograde (red) and retrograde (green) kymograph plots upon which the data are based. Horizonal scale bars represent 1μm and vertical scale bars indicate a 5 second interval.

bioRxiv preprint doi: https://doi.org/10.1101/2025.10.23.684198; this version posted October 24, 2025. The copyright holder for this preprint (which was not certified by peer review) is the author/funder, who has granted bioRxiv a license to display the preprint in perpetuity. It is made available under aCC-BY 4.0 International license.

Supplemental Table S1 List of strains used

STRAIN	GENOTYPE
BT40	*em16 [CFH-1::eGFP]; emIs5 [dat-1p::NPHP-2::mCherry+rol-6(su1006)]*
BT41	*emIs5 [dat-1p::NPHP-2::mCherry+rol-6(su1006)]; oq118 [ARL-13::mNG]*
BT42	*cfh-1(em14); emIs5 [dat-1p::NPHP-2::mCherry+rol-6(su1006)]; oq118 [ARL-13::mNG]*
	myEx1[Posm-5::OSM-5::GFP + pRF4]; xbx-1(cas502[xbx-1::tagRFP])
BT62	*tep-1(ok2874); myEx1[Posm-5::OSM-5::GFP + pRF4]; xbx-1(cas502[xbx-1::tagRFP])*
BT66	*myEx10[Pche-11::CHE-11::GFP + pRF4]; xbx-1(cas502[xbx-1::tagRFP])*
BT72	*dpy-5(e907); sEX14690[tep-1p::GFP+ dpy-5]; ns108[ptr-10p::myrRFP]*
BT73	*em19[TEP-1::mNG]; ns108[ptr-10p::myrRFP]*
BT74	*em19[TEP-1::mNG]; emEX[grl-2p::mApple+rol-6(su1006)]*
BT75	*em19[TEP-1::mNG]; syb7720 [VAB-9::wrmScarlet]*
BT76	*em19[TEP-1::mNG]; emEX[bbs-8p::XBX-1::tag::RFP]*
BT77	*em19[TEP-1::mNG]; emIs5[dat-1p::NPHP-2::mCherry+rol-6(su1006)]*
BT78	*em19[TEP-1::mNG]; emEX[bbs-8p::NPHP-2::mCherry+rol-6(su1006)]*
BT79	*em16[CFH-1::eGFP]; em20[TEP-1::wrmScarlet]*
BT80	*em16[CFH-1::eGFP]; emEX[bbs-8p::NPHP-2::mCherry+rol-6(su1006)]*
BT81	*tep-1(em18); em16 [CFH-1::eGFP]; emIs5 [dat-1p::NPHP-2::mCherry+rol-6(su1006)]*
BT82	*tep-1(ok2874); em16 [CFH-1::eGFP]; emIs5 [dat-1p::NPHP-2::mCherry+rol-6(su1006)]*
BT83	*cfh-1(em14); tep-1(ok2874); emIs5 [dat-1p::NPHP-2::mCherry+rol-6(su1006)]*
BT84	*tep-1(ok2874); em16 [CFH-1::eGFP]; emIs5 [dat-1p::NPHP-2::mCherry+rol-6(su1006)] +emEX[tep-1p::tep-1+unc-122p::RFP]*
BT85	*tep-1(ok2874); em16 [CFH-1::eGFP]; emIs5 [dat-1p::NPHP-2::mCherry+rol-6(su1006)] +emEX[ptr-10p::tep-1gpi+unc-122p::RFP]*

bioRxiv preprint doi: https://doi.org/10.1101/2025.10.23.684198; this version posted October 24, 2025. The copyright holder for this preprint (which was not certified by peer review) is the author/funder, who has granted bioRxiv a license to display the preprint in perpetuity. It is made available under aCC-BY 4.0 International license.

BT86	*tep-1(ok2874); em16 [CFH-1::eGFP]; emIs5 [dat-1p::NPHP-2::mCherry+rol-6(su1006)] +emEX[ptr-10p::tep-1s+unc-122p::RFP]*
BT87	*tep-1(ok2874); em16 [CFH-1::eGFP]; emIs5 [dat-1p::NPHP-2::mCherry+rol-6(su1006)] +emEX[bbs-8p::tep-1gpi+unc-122p::RFP]*
BT88	*tep-1(ok2874); em16 [CFH-1::eGFP]; emIs5 [dat-1p::NPHP-2::mCherry+rol-6(su1006)] +emEX[bbs-8p::tep-1s+unc-122p::RFP]*
BT89	*emEX[bbs-8p::NPHP-2::mCherry+rol-6(su1006)]*
BT90	*tep-1(ok2874); emEX[bbs-8p::NPHP-2::mCherry+rol-6(su1006)]*
BT91	*cfh-1(em14); emEX[bbs-8p::NPHP-2::mCherry+rol-6(su1006)]*
BT92	*cfh-1(em14); tep-1(ok2874); emEX[bbs-8p::NPHP-2::mCherry+rol-6(su1006)]*
BT93	*tep-1(ok2874); emIs5 [dat-1p::NPHP-2::mCherry+rol-6(su1006)]; oq118 [ARL-13::mNG]*
BT94	*tep-1(ok2874); che-3(cas443[gfp::che-3]); xbx-1(cas502[xbx-1::tagRFP])*
BT95	*tep-1(ok2874); myEx10[Pche-11::CHE-11::GFP + pRF4]; xbx-1(cas502[xbx-1::tagRFP])*
GOU2162	*che-3(cas443[GFP::che-3]); xbx-1(cas502[xbx-1::tagRFP])*

Figure S1

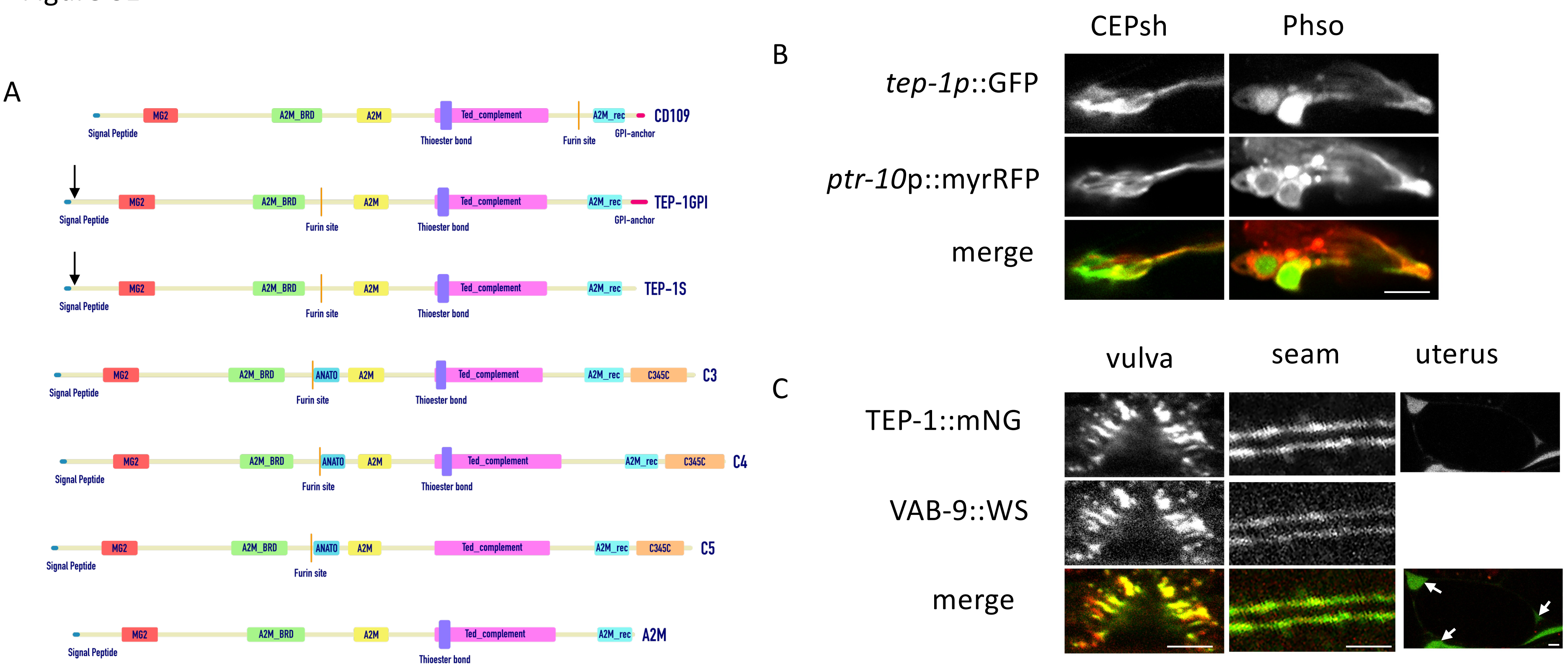
A
Signal Peptide
MG2
A2M_BRD
A2M
Ted_complement
Thioester bond
Furin site
A2M_rec
GPI-anchor
CD109
TEP-1GPI
TEP-1S
ANATO
C345C
C3
C4
C5
A2M
B
CEPsh
Phso
tep-1p::GFP
ptr-10p::myrRFP
merge
C
vulva
seam
uterus
TEP-1::mNG
VAB-9::WS
merge

Figure S2

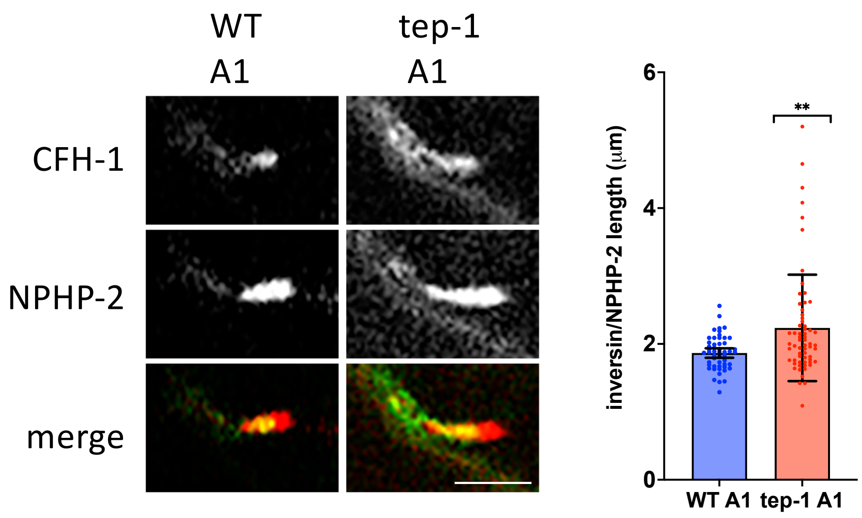

WT
A1
tep-1
A1
CFH-1
NPHP-2
merge
6
4
2
0
inversin/NPHP-2 length (μm)
**
WT A1
tep-1 A1

Figure S3

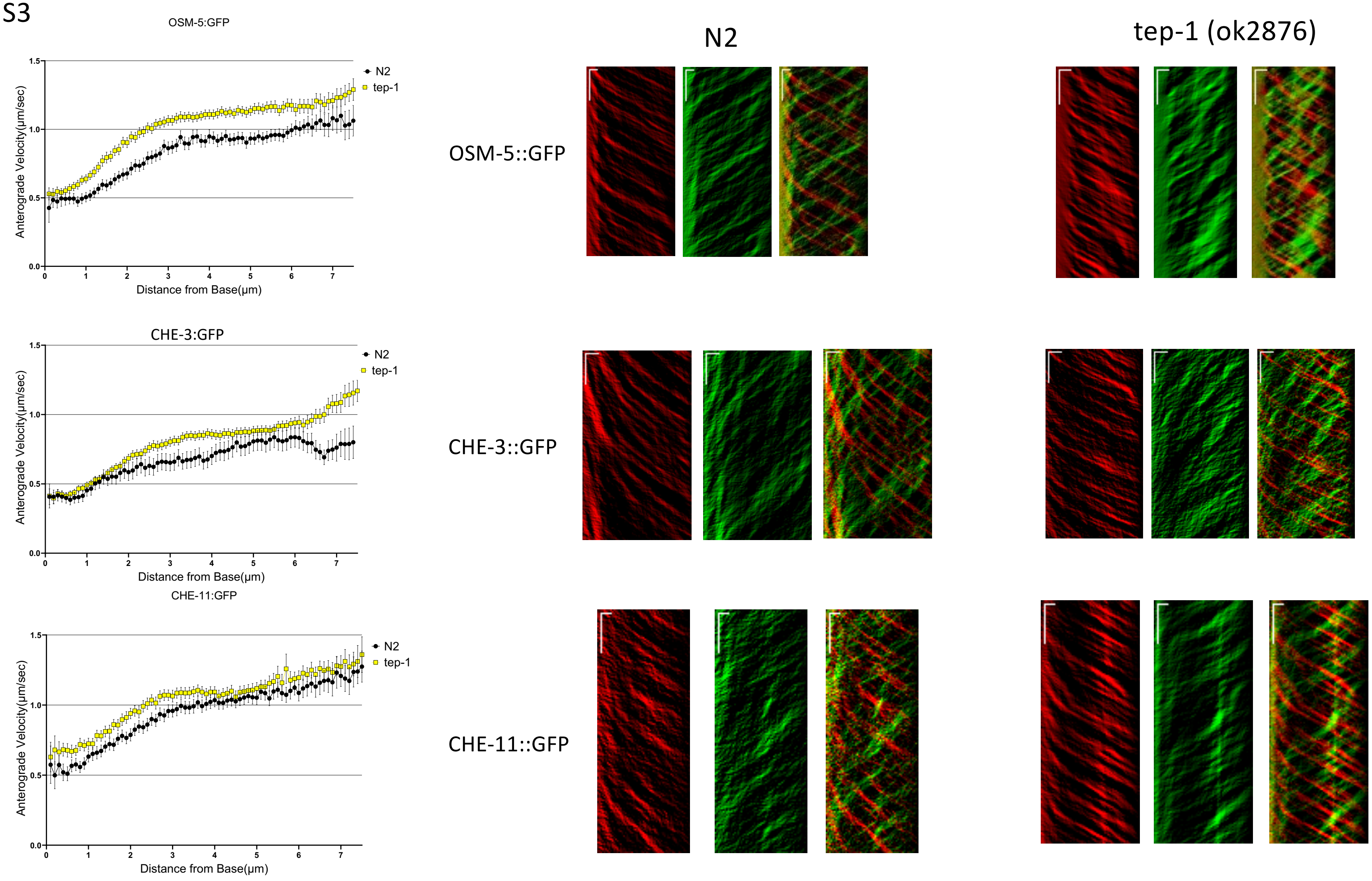

OSM-5:GFP
Anterograde Velocity(μm/sec)
Distance from Base(μm)
N2
tep-1
CHE-3:GFP
Anterograde Velocity(μm/sec)
Distance from Base(μm)
N2
tep-1
CHE-11:GFP
Anterograde Velocity(μm/sec)
Distance from Base(μm)
N2
tep-1
N2
tep-1 (ok2876)
OSM-5::GFP
CHE-3::GFP
CHE-11::GFP
